# Sparse Adversarial Video Attacks via Superpixel-Based Jacobian Computation

**DOI:** 10.3390/s22103686

**Published:** 2022-05-12

**Authors:** Zhenyu Du, Fangzheng Liu, Xuehu Yan

**Affiliations:** College of Electronic Engineering, National University of Defense Technology, Hefei 230037, China; liufangzheng17@nudt.edu.cn (F.L.); yanxh17@nudt.edu.cn (X.Y.)

**Keywords:** adversarial examples, video classification, temporal sparsity, spatial sparsity

## Abstract

Adversarial examples have aroused great attention during the past years owing to their threat to the deep neural networks (DNNs). Recently, they have been successfully extended to video models. Compared with image cases, the sparse adversarial perturbations in the videos can not only reduce the computation complexity, but also guarantee the crypticity of adversarial examples. In this paper, we propose an efficient attack to generate adversarial video perturbations with large sparsity in both the temporal (inter-frames) and spatial (intra-frames) domains. Specifically, we select the key frames and key pixels according to the gradient feedback of the target models by computing the forward derivative, and then add the perturbations on them. To overcome the problem of dimensional explosion in the video, we introduce super-pixels to decrease the number of pixels that need to compute gradients. The proposed method is finally verified under both the white-box and black-box settings. We estimate the gradients using natural evolution strategy (NES) in the black-box attacks. The experiments are conducted on two widely used datasets: UCF101 and HMDB51 versus two mainstream models: C3D and LRCN. Results show that compared with the state-of-the-art method, our method can achieve the similar attacking performance, but it pollutes only <1% pixels and costs less time to finish the attacks.

## 1. Introduction

The development of DNNs brings significant convenience to people’s lives. However, recently, researchers have found that DNNs are vulnerable to adversarial examples [1]. The works have shown that an image with small perturbations can fool a classification system trained by DNNs. These images with imperceptible perturbations are called adversarial examples AEs.

The low-cost adversarial examples will make the DNNs return the wrong output, and they thus bring great damage and harm to the applications based on DNNs. Adding perturbations to the road signs would cause the auto-driving system to make a wrong decision [2]. Wearing adversarial glasses [3] or hats [4] would enable a person to pretend to be someone else when passing the surveillance systems. Dressing in adversarial T-shirts would make criminals disappear from the surveillance systems [5]. These AEs cause great inconvenience and harm to people’s lives.

The concerns about the video models’ security cause the focus of AEs to turn to adversarial video examples (AVEs). Recently, the key applications based on video classification models have begun to be applied to some critical systems. Those AI systems are directly related to personal safety and property security. For example, they are widely used in the fields of smart home [6], automatic driving [7], elderly care [8], and property protection. However, the harm brought by AEs would cause a great threat to those critical systems. Therefore, it is crucial to study the adversarial examples of video models and thus further improve the robustness of those models.

According to the number of perturbations added to the inputs, the current attacks by AVEs can be divided into two classes according to their settings. One is the sparse AVEs, and the other is dense AVEs. The sparse AVEs only add perturbations on several frames [9], not all the frames. However, the dense AVEs add perturbations on each frame among a video (similar to [10,11]). At the beginning of the research on AVEs, people were focused on breaking through the boundary between AVEs and AEs. They tried to convert the AEs generation methods, such as FGSM [1], and other methods [12,13] to AVEs. However, these methods have lower fooling rates. Then, some researchers generated more effective AVEs based on the video feature (such as [10,11]). However, these methods cost great resources to generate one AVE because they need to add the perturbations on each frame among a video. Therefore, the work [9] proposes a method that only adds perturbations on several sparse frames to decrease the number of perturbed frames and acquire a better performance on the fooling rate.

In the sparse AVEs, they use spatial sparsity and temporal sparsity to define the number of perturbed frames and pixels. Specifically, temporal sparsity means the proportion of clean frames versus all frames of a video. The spatial sparsity means the proportion of clean pixels versus all pixels of a video. A higher temporal sparsity and spatial sparsity mean fewer frames and pixels being perturbed. The detailed definitions of them are described in the following section.

Compared to the dense AVEs, the sparse AVEs avoid the redundancy of the adversarial perturbations and have the following advantages: (1) Sparse video attack can reduce the computational complexity. Sparse video attack only adds perturbations on several frames and has a lower total value of adversarial perturbations. There is no need to take the computational cost to generate complex perturbations. According to our experimental results, our sparse attack costs less time to generate adversarial videos compared with other sparse attacks; (2) Sparse video attack can improve the imperceptibility to human observers. Our sparse attack has higher spatial sparsity and temporal sparsity than other sparse adversarial video attacks. This means that it only needs to perturb fewer pixels of videos to generate adversarial videos. Meanwhile, for human observers, it has better invisibility; (3) Sparse video attack gives a better interpretation of adversarial video attack. The perturbation positions reveal which frame and which part of that frame are important but also vulnerable for the prediction by the video classifier; (4) Sparse video attack can decrease the queries of adversarial video attack under black-box settings. Sparse video attack generates adversarial videos in a low-dimensional manifold. It decreases the high dimension of videos. Compared to dense attacks, the more the sparsity of the adversarial video attack, the fewer queries it needs to query the results from the black-box models.

**Figure 1:**
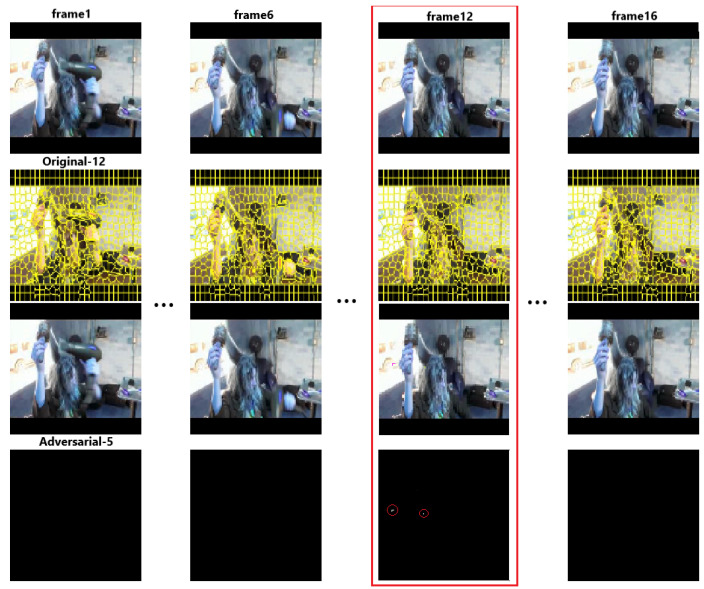
Examples of adversarial frames generated by our algorithm with only one frame disturbed. The first line is the original image, the second line is the image with super-pixels, the third and fourth lines are the adversarial image and difference, respectively. To see clearly, the noise is circled by red circle. The original and adversarial labels are signed under the first and third line. The label 12 is “BlowDryHair” and 5 is “BandMarching”.

However, the current sparse video attack is not sparse enough. First, they only focus on generating AVEs with temporal sparsity and ignore the spatial sparsity. Specifically, they only consider how to decrease the number of perturbed frames and do not consider how to decrease the number of perturbed pixels among the perturbed frames. However, different from images, the video has two-dimensional features: the temporal and the spatial features. Therefore, it is crucial to consider the temporal sparsity and spatial sparsity simultaneously. Second, the temporal sparsity is not enough. Current sparse AVEs still need to perturb nearly half of the video frames to acquire a successful attack. However, with the development of video classification models, they need to input more frames to obtain better performance, such as increasing inputs from 16 frames as input to 48 frames extracted from a video. Therefore, the cost would significantly increase, and current sparse AVEs still need great cost to generate successful AVEs. Therefore, there still is a gap between the temporal sparsity and the real requirements, and the key point in a sparse attack is working out how to increase the temporal and spatial sparsity of an adversarial video simultaneously as much as possible. Figure 1 shows one of the AVEs generated by our algorithm. In Figure 1, the last line is the adversarial perturbations that we add to the original videos. We can only perturb one frame and several pixels to generate the AVEs with better spatial sparsity and temporal sparsity.

Therefore, in this paper, we present a method to generate sparse adversarial videos, which can significantly improve both the temporal and spatial sparsity at the same time. We are motivated by the physical meaning of the forward derivative. According to its definition, the forward derivative of each pixel can reflect the contribution of that pixel to the output of video models. Thus, we calculate the forward derivative of the frames. It can also reflect the contribution of that frame to the output. We select the key frames and key pixels according to their forward derivative, respectively, and then only add perturbations on the selected key frames and pixels. The method of selecting key frames and pixels can significantly improve the temporal sparsity and spatial sparsity.

However, compared with image data, video data have the problem of dimension explosion when computing the forward derivative. To solve this problem, we introduce the super-pixels [14], and compute the gradients based on the super-pixels, i.e., the pixels within one super-pixel share the same gradients. In this way, we can reduce the number of pixels to be computed.

Moreover, different from other adversarial generation methods that can only adapt to one setting, either the white-box setting or the black-box setting, we also devise a framework to generate adversarial examples under the two settings. In the white-box video attack, the attacker knows all the knowledge of a model, while in the black-box video attack, the attacker can only access the model’s outputs.

Specifically, in the white-box attack, we first use the super-pixels to reduce the dimension of a video and compute each super-pixel’s forward derivative as the contribution. Then we sum that value within a frame as the contribution of that frame and search for the key frames, key super-pixels, and key pixels by ranking those contributions. Lastly, we can acquire the adversarial videos by only perturbing these key pixels. We can find the closest adversarial videos to the original sample, which has the lowest number of disturbed pixels, through this method.

In the black-box attack, we cannot directly calculate the contribution of frames or pixels due to the lack of models’ details. Therefore, we choose to estimate the forward derivative based on natural evolution strategy (NES) [15]. However, this value’s accuracy and total resource consumption are directly proportional to the number of pixels needed to be calculated. Therefore, different from the white-box attack, we first select the key frames by the output of every single frame to reduce the dimension of the video and then use super-pixels to reduce the dimension of the key frames, which can decrease the number of objects needed to be estimated to as few as possible. After that, we select the key pixels by the estimated contribution. Finally, we add perturbations on the selected key pixels. Using iterative computations, we will generate an adversarial video with the smallest perturbations in a short time.

Our major contributions can be summarized as follows:

- We propose a novel method to generate adversarial videos with large sparsity in both the temporal and spatial domains. It adds perturbations only on the key pixels of the key frames and finally generates an adversarial video with fewer pixels disturbed in a short time.
- We introduce super-pixels to solve the dimension explosion problem that exists in attacking video data. This method can decrease the number of pixels whose gradients need to be computed, and thus improve the efficiency of generating adversarial examples.
- Our algorithm can work in different two settings: the white-box attack and the black-box attack. We test its attacking performance against two mainstream models on two public datasets. Results show that compared with the state-of-the-art video attacking methods, our method can achieve a similar attacking performance, but it only pollutes <1% pixels and costs less time.

The rest of this paper is organized as follows: In Section 2, we briefly review the related work. In Section 3, we describe our algorithm in detail. In Section 4, we present our experimental results and compare them with other methods. Finally, we conclude our paper in Section 5.

## 2. Related Work

### 2.1. Adversarial Attack on Image Models

Many works have focused on generating adversarial samples for images in recent years. These works can be divided into two categories due to their attacking condition: white-box and black-box attacks.

White-box attack assumes that the structure and parameters of a target model are known to the attacker. In white-box attack, it is easy to perform attacks according to the gradient of objective function of attack or the forward derivative of model computed via backpropagation. Various types of attacks have been proposed. For example, L-BFGS [16], fast gradient sign method (FGSM) [1], deep fool [7], Jacobian-based saliency map attack (JSMA) [17], basic iterative method (BIM) [12], C&W attack [18], and universal attack [19]. In the JSMA attack, the attackers use the forward derivatives of input to sort the contributions of each element of inputs to the output. Specifically, the forward derivative refers to the partial derivative of each output of the last layer of the neural network to each input, as shown in Equation (1).

(1) $$\nabla F\left(X\right)=\frac{\partial F(X)}{\partial X}=\frac{\partial f_{j}\left(X\right)}{\partial x_{i}}{}_{i\in 1\cdots N,\;\;j\in 1\cdots M}$$

In Equation (1), $F_{j}\left(X\right)$ represents the score of the output of the classifier *F* on the *j*th category when inputting *X*, and $x_{i}$ represents the *i*th input feature. The forward derivative gives an instruction of the input that has the greatest impact on the specific output of the classifier. Therefore, adding perturbations to that input can significantly influence the output and help to generate effective AEs.

Contrarily, the black-box setting remains the model with information unknown to attacker, which makes generation of adversarial samples more challenging. Existing works in this setting include zeroth-order optimization (ZOO) [20], autoencoder-based zeroth-order optimization method (AutoZOOM) [21], decision-based attack [22], and opt-attack [23].

These adversarial attacks can generate AEs for image models, and they would bring much harm to the systems based on the models. For example, the work [8] proposes a dataset to help to estimate the risk of falls of the people. If generating the AEs for the models based on that dataset, researchers cannot give an exact prediction of the fall, and bring risk to the older people. In addition, the work [24] proposes a software engine to simulate the links delivering a video flow from a video source. If generating the AEs for the video flow, it would make the videos contaminated and risk the security of the links.

### 2.2. Adversarial Attack on Video Models

In 2019, some works focused on how to generate adversarial samples of video models. In a white-box attack, an $l_{2,1}$-norm regularization-based optimization is the first method to compute the sparse adversarial perturbations for video recognition [9]. After that, the work [10] utilized generative adversarial networks (GANs) to generate 3D universal perturbation offline. In a black-box attack, the work [11] achieves good performance in generating adversarial videos based on tentative perturbations and partition-based rectifications. Furthermore, the work [25] heuristically searches a subset of frames and adds perturbations only on those frames separately. The works mentioned above needed too many pixels modified to generate an adversarial video, and the spatial sparsity is too small.

Unlike the algorithms mentioned above, our method computes key frames based on forward derivatives and only adds perturbations on key pixels among the key frames to generate adversarial videos. Our method can generate adversarial videos with fewer pixels perturbed. Finally, we successfully implement it both in black-box and white-box settings.

## 3. Methodology

**Figure 2:**
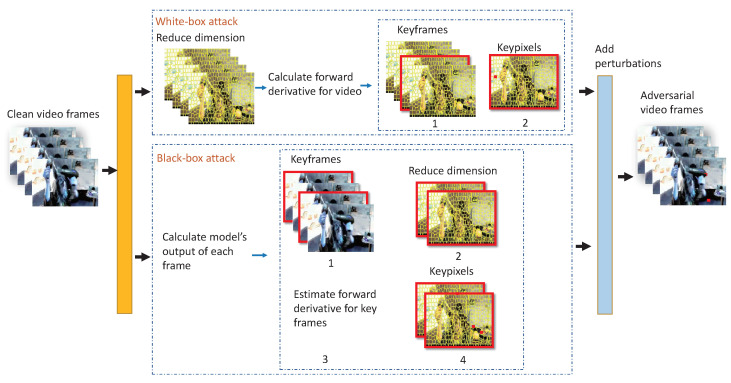
The overall framework. We illustrate the frameworks in the white-box attacks and black-box attacks, respectively. The top part is the white-box attack pipeline and the bottom part is the black-box attack pipeline.

Our sparse attack of video models can be implemented both in white-box and black-box conditions, and generate the target and non-target AVEs in the two conditions. Figure 2 overviews the proposed method. The algorithm is divided into two parts: one is the white-box attack shown at the top and the other one is the black-box attack at the bottom. In the white-box attack, we can access the structure and parameters of the threat classifier *F*, but in the black-box attack, the only knowledge of the model we have is the outputs.

In the the white-box setting, it calculates the super-pixels to reduce dimension of input video, and then it calculates the forward derivative of each super-pixel as the contribution of video to the output. According to the forward derivatives of each super-pixel, it can calculate the contribution of the frames when all derivatives in total are among a frame. The forward derivative is the contribution of a frame to the output. Then, we construct the saliency map to show the different inputs that have the greatest impact on the specific output of the classifier. Through the saliency map, we select the key frames, key super-pixels, and key pixels. Finally, it adds adversarial perturbations to the key pixels to generate effective AVEs. Through this method, we can generate the AVEs close to the original example.

In the black-box attack, we first select the key frame according to the output of each frame. Then, it uses super-pixels to reduce the dimension of the key frame, and due to the parameters being inaccessible, we estimate the forward derivative based on NES instead. Finally, we select the key pixels according to the forward derivative and add adversarial perturbations to the key pixels.

Above all, the key differences of the algorithm between the two conditions are how to acquire the forward derivative of the super-pixel among a video and the node for dimension reduction. We detail the difference in the following section.

### 3.1. White-Box Attack

Denotation: In this setting, we define the classifier function of the threat model as *F*, a clean video input as $X\in R^{T\times W\times H}$, where T,W,andH denote the number of frames, frame width and frame height, respectively. The ground-truth label of *X* is defined as y∈{1,⋯,C}, where *C* is the number of classes. $X=\{x_{i}|i=1,\cdots ,T\}$, $x_{i}\in R^{W\times H}$ is the *i*-th frame of *X*. We use $\psi_{ij}$ as the *j*-th super-pixel of frame $x_{i}$, and $p_{ijk}$ as the *k*-th pixel within the $\psi_{ij}$. Thus, $x_{i}=\left\{\psi_{ij}|j=1,\cdots ,M\right\}$, and $\psi_{ij}=\left\{p_{ijk}|k=1,\cdots ,K\right\}$. An adversarial video $X_{adv}\in R^{T\times W\times H}$ makes $F(X_{adv})\neq y$ in the non-target attack while $F(X_{adv})=g$ in the target attack, where *g* is the target label. If a frame $x_{i}$, a pixel $p_{ijk}$, and a super-pixel $P_{ij}$ are labeled as $x_{i}^{*}$, $p_{ijk}^{*}$, and $\psi_{ij}^{*}$, it means that they are the key frame, key pixel, and key super-pixel, respectively. We also define $X^{*}$ as the set of key frame and $P^{*}$ as the set of key super-pixels.

The main flow of the algorithm under the white-box setting is

- Determine the label of attack target.
- Reduce dimension for the video.
- Compute forward derivative and saliency map.
- Search for key frames and key pixels.
- Add perturbations.

#### 3.1.1. Determine the Label of Attack Target

The first step of the algorithm is to determine the label of attack target, which can be specified by the attacker. When it is the non-target attack, the algorithm can automatically calculate the category closest to the original sample as the target category *g*. Compared with selecting a label as target randomly, it can cost the minimum resources to generate the AVEs that can make the model give a wrong label. The equation is Equation (2). The loss is the cross-entropy function.

(2) $$\begin{array}{cc} \; & g=\underset{i\neq y}{\arg\min\mathrm{loss}(i,y)}\\ \; & \;s.t.\;y=F(X) \end{array}$$

#### 3.1.2. Reduce Dimension for the Video

Super-pixels algorithms group pixels into perceptually meaningful atomic regions which can be used to replace the rigid structure of the pixel grid. They capture image redundancy and greatly reduce the complexity of subsequent image processing tasks [14]. We first reduce dimension of a video by introducing SLIC, a state-of-the-art super-pixels algorithm. It is used to adapt *K*-means clustering to generate super-pixels. Ideally, after this process, if it combines the ε pixels around one pixel in the frame into the same pixel, the number of pixels whose derivative need to be computed will decrease from T×W×H to (T×W×H)/ε. We denote the video after super-pixel calculation as Ψ, Ψ=SLIC(X).

#### 3.1.3. Compute Forward Derivative and Saliency Map

After reducing the dimension of the video, the algorithm needs to acquire the contribution of each super-pixel to perform the selection step.

We thus compute the forward derivative of that processed video Ψ, under the output of classifier F(Ψ) as $\nabla {}_{\Psi }F\left(\Psi \right)$. $\nabla {}_{\Psi }F\left(\Psi \right)$ is a tensor, which describes the contribution of each super-pixel among that video to the score of classifier under all *C* classes. Each element $\lambda_{\mathrm{cij}}$ of that cube Λ is the first-order partial derivative of *j*th super-pixel $\psi_{ij}$ within *i*th frame $x_{i}$ under the *c*th class. That value is positively correlated to the contribution of that super-pixel to the score of the current class. We compute $\nabla {}_{\Psi }F\left(\Psi \right)$ by Equation (3).

Specifically, according to the physical meaning of the forward derivatives [26], the forward derivative tells us which input regions are unlikely to yield minor perturbations. When the forward derivative of super-pixel $\psi_{ij}$ bigger than 0, that is $\lambda_{cij}>0$, it means that when adding minor perturbations to that super-pixel, the output of target class $F_{c}$ would increase. Meanwhile, the bigger the $\lambda_{cij}$, the more influence the super-pixel gives to the output of the model. Therefore, the value of Equation (3) shows the influence of super-pixels.

(3) $$\lambda_{cij}=\frac{\partial F_{c}\left(\Psi \right)}{\partial \psi_{ij}}$$

The $F_{c}\left(\Psi \right)$ means the output on *c*th class of that video after being a reduced dimension. According to that cube, we can acquire the forward derivative of each super-pixel corresponding to *C* classes. Specifically, in the classification model, the final output of the classification model depends on the output scores of the video in all *C* classes. Therefore, in order to measure the criticality of a super-pixel, the algorithm needs to comprehensively consider both its contribution to the target class and its contribution to other categories. If a super-pixel can make a positive contribution to the target category but a negative contribution to other categories, that super-pixel would play a major role when it is disturbed. Therefore, in order to generate more effective AVEs, we need to find a super-pixel with the positive value of the forward derivative of the target class but a negative value of the other class. To successfully search for the super-pixels with those features, we thus construct the saliency map S by the forward derivative computed before as Equation (4).

The saliency map S is a sparse array, that is, its element has effect value only when a super-pixel has a positive value of the forward derivative of the target class while a negative value of the other classes, but in other cases, the value is 0. We call a super-pixel with a valid value an effective super-pixel, which means that super-pixel $\psi_{ij}$ will contribute more to generate the adversarial video than other super-pixels. Adding appropriate perturbations to that super-pixel can move the video to the target class more easily. Equation (4) shows the saliency map.

(4) $$S_{ij}=\left\{\begin{array}{cc} \;\;\;\;\;\;\;0, & otherwise\\ \lambda_{(c=g)ij}\sum_{c\neq g}|\lambda_{cij}|, & \mathrm{if}\;\lambda_{(c\neq g)ij}>0\;\;\&\&\;\;\lambda_{(c=g)ij}>0 \end{array}\right.$$

#### 3.1.4. Search for Key Frames and Key Pixels

With the help of $S_{ij}$, the algorithm can measure the effectiveness of each super-pixel. However, suppose we filter the key super-pixels directly in the whole video by $S_{ij}$. In that case, it may cause the final generated perturbations to spread among all the frames of a video sample, and it will result in a very low temporal sparsity of the generated adversarial videos. Therefore, to improve temporal sparsity, we should control the number of disturbing frames. We should first filter the key frames $x_{i}^{*}$ and then select the key super-pixels ${\psi_{ij}}^{*}$ among those frames.

In order to search for the key frames, the algorithm calculates the saliency map for each frame $S_{i}$ by Equation (5). Similar to the physical meaning of $S_{ij}$, $S_{i}$ means the criticality of each frame of the input video. Therefore, the algorithm selects a frame $x_{i}$ with the maximum $S_{i}$ as the key frame $x_{i}^{*}$ and a super-pixel with the largest saliency map value $S_{ij}$ as the key super-pixels $\psi_{ij}^{*}$ of $x_{i}^{*}$.

(5) $$\begin{array}{cc} \; & x_{i}^{*}=\underset{1\leq i\leq T}{\arg\max}\left(S_{i}\right)\\ \; & s.t.S_{i}=\sum_{j=1}^{M}S_{ij} \end{array}$$

However, in that part, we should solve two problems: one is how to select more key frames and super-pixels effectively; the other is that adding perturbations to the key super-pixels brings redundancy. We show the details in the following words.

First, it does not work well when only selecting one key frame and one key super-pixel. In most cases, for an effective attack, we should choose more than one key frame and key super-pixels to add perturbations to them. It means that adding perturbations on a single key frame or key super-pixel alone is not enough to cause models to misclassify the modified input. Therefore, we define *n* as the number of key super-pixels selected in each iteration to make the attack successful. There is no need to set a variable to define the number of key frames such as *n*, though we also need to search more than one key frame. The reason is that the maximum $S_{i}$ would change with different *n*. Therefore, the index of key frame will change automatically with the key super-pixels in the experiment.

We rank the saliency map of the selected key frame, and select the super-pixels with top *n* values to construct the set of key super-pixels $P^{*}$ of key frame $x_{i}^{*}$, and when the algorithm is to the end, we collect the index *i* of key frame $x_{i}^{*}$ selected in each iteration and integrate them as the set of key frames $X^{*}$.

Second, it brings redundancy if directly adding perturbations to the key super-pixels. The super-pixels contain lots of real pixels of a video. If adding perturbations on all pixels belonging to super-pixels directly, the perturbations will be more perceptible to human eyes. Moreover, these pixels do not share the same forward derivative in the real video, so their real contributions to output are not the same. Therefore, adding perturbations on key super-pixels will cause pixels waste. We select only *n* pixels of those super-pixels as key pixels $p_{ij}^{*}$, and only add perturbations on these key pixels.

Lastly, the algorithm should set an appropriate value of *n*. When *n* is small, the algorithm will drop into an “endless loop” quickly. The reason is that the perturbed key pixels reach the boundary value and cannot be modified anymore, so the algorithm will compute the same saliency map, select the same key frame and the same key super-pixels in each iteration, and finally still modify the same pixels of those super-pixels.

To solve this problem, one trick is to change *n* into a larger one and the other is to change the boundary value of each pixel into a larger one. However, if we choose the second trick, perturbations of key pixels will become larger, which leads to larger total distortion of key frame so that more perceptible perturbations are shown in an adversarial video. Considering that the number of disturbed pixels and perturbations of each pixel are contrary variables, we should find a balance between them under the constraints of a successful attack. In our algorithm, we dynamically change *n* to find that balance, keeping the pixel boundary value maxp,minp unchanged. Specially, considering the global time consumption, we set *n* as a small value in the beginning of the algorithm and gradually increase its value when all the key pixels reach to the boundary, and the algorithm will double *n* when it falls into an “endless loop”.

Above all, in the process of selecting the key super-pixel, we first rank the saliency map S and initially set n=1, and then select *n* key frames, key super-pixels, and key pixels. If the perturbations are not enough to make a successful attack, the algorithm will double *n* and then repeat the process.

#### 3.1.5. Add Perturbations

Finally, we add perturbations to these key pixels within key frames by Equation (6). The perturbations should be able to move the video as close as possible to the target class in target attack or as far as possible to the ground class in non-target attack. Therefore, the direction of perturbations should be the same as the sign of forward derivative of that pixel under the target class, which is $sgn(\frac{\partial F_{g}\left(X\right)}{\partial p_{ijk}^{*}})$. η is the basic value of disturbance, means the one added to each key pixels in each iteration, and the $sgn(\frac{\partial F_{g}\left(X\right)}{\partial p_{ijk}^{*}})$ is the same as $sgn(\frac{\partial F_{g}\left(X\right)}{\partial \psi_{ij}^{*}})$, the direction of the super-pixel that the key pixel belongs to.

(6) $${p_{ijk}^{*}}^{\prime }=p_{ijk}^{*}+\eta sign\left(\frac{\partial F_{g}\left(X\right)}{\partial p_{ijk}^{*}}\right)$$

The whole algorithm in the target attack is summarized in Algorithm 1, where maxe is the maximum iteration number, and maxp and minp are the boundary value of pixels in the video.
**Algorithm 1** Crafting the White-Box Attack in the Target Mode**Input**X,F(·)**Input Parameters**g,C,y,maxe,n,η,maxp,minp**Output**$X_{adv}$1:  Let $X_{adv}\leftarrow X$, $X^{*}=\left\{\right\}$, iter=1.2:  Set the target label g≠y.3:  Reduce the dimension of *X* using SLIC: $X_{\mathrm{adv}}\leftarrow SLIC\left(X_{\mathrm{adv}}\right)$.4:  **while** $F(X_{\mathrm{adv}})\neq g$ and iter≠maxe **do**5:     Compute forward derivative $\nabla_{X_{adv}}F\left(X_{\mathrm{adv}}\right)$ by Equation (3).6:     Construct $S_{ij}$ by Equation (4).7:     Compute $S_{i}$ by Equation (5).8:     $x_{i}^{*}=\underset{1\leq i\leq T}{\arg\max}\left(S_{i}\right)$9:     **if** $x_{i}^{*}\notin X^{*}$ **then**10:       Add $x_{i}^{*}$ to the set of key frame $X^{*}$.11:   **end if**12:   Rank $S_{ij},j\in \left[1,M\right]$.13:   Select super-pixels with top *n* value of $S_{ij}$ as key super-pixels $\psi_{ij}^{*}$ of key frame $x_{i}^{*}$.14:   Select *n* key pixels $p_{ijk}^{*},k\in \left[1,n\right]$ within $\psi_{ij}^{*}$ of $x_{i}^{*}$.15:   $p_{ijk}^{*}=p_{ijk}^{*}+\eta sgn\left(\frac{\partial F_{g}\left(X\right)}{\partial p_{ijk}^{*}}\right)$.16:   **if** all the value of key pixels ∉[minp,maxp] **then**17:       n←n×2.18:   **end if**19:   iter←iter+1.20:**end while**21:**return**$X_{adv}$

#### 3.1.6. Analysis of the Algorithm under the White-Box Setting

This section analyzes the computational complexity and the implementation cost of the AVEs generation method under the white-box setting. First, according to the work [14], the computational complexity of the algorithm SLIC is O(N), where *N* is the number of pixels needs to calculate super-pixels. In that algorithm, under the white-box setting, it needs to calculate super-pixels for all the videos. Therefore, N=T×W×H, and thus the complexity is O(T×W×H). In addition, in the algorithm’s loop, the key step is computing the forward derivatives of the video. The complexity of that step is also O(n), where *n* is the number of objects needed to calculate forward derivatives. It is the number of super-pixels of the video. Therefore, the complexity of computing the forward derivatives is O((T×W×H)/ε). Because the maximum number of the loop is maxe, the worst complexity is O(maxe×(T×W×H)/ε), and the best complexity is O((T×W×H)/ε).

However, according to that, the algorithm uses DNNs to compute related parameters and the parameters of the DNNs model are too many to be used to calculate the complexity exactly. Therefore, in our paper, we use the metric time to evaluate the time complexity of that algorithm. The detailed results are shown in Section 4.1.3.

### 3.2. Black-Box Attack

In the black-box setting, we can only access the output of a video model. Therefore, different from the white-box attack, we estimate the forward derivative of the model based on NES [15] instead of computing it directly.

However, the computational cost positively correlates to the number of super-pixels that need to be estimated. In a black-box attack, if we use the same method of white-box attack that selects the key frame according to the forward derivative, we should estimate the forward derivative of all the super-pixels of a video. That step is time-consuming. In addition, the more the derivatives need to estimate, the lower their accuracy. Therefore, it is necessary to reduce the number of estimated objects.

The main flow of the algorithm under the white-box setting is

- Determine the label of attack target.
- Search for key frames.
- Compute forward derivative and reduce dimension for the key frames.
- Estimate the forward derivative and saliency map.
- Search for key pixels.
- Add perturbations.

The difference between the flow of white-box setting and black-box setting are sections “search for key frames”, “estimate the forward derivative and saliency map”, and “search for key pixels”. We thus detail the difference as follows.

#### 3.2.1. Search for Key Frames

We know that the key frames contribute more to the output than ordinary frames. Therefore, we compute the output of the target class *g* of each frame. The key frame will take the largest one of that value. Specifically, we define M, a length *T* vector, where all elements of it are 0 initially. Then, we change $i_{th}$ element of M from 0 to 1, then compute the $F_{g}\left(X\times M\right)$ as the contribution of that frame $x_{i}$ to the target class. We construct a list F to record that value, where each element F of it is the contribution of frame $x_{i}$. Finally, the frame with the largest value in the list is the key frame $x_{i}^{*}$ of that iteration. It can be shown as Equation (7).

(7) $$\begin{array}{cc} \; & F_{i}=F_{g}\left(X\times M\left(M\left[i\right]=1\right)\right)\\ \; & x_{i}^{*}=\underset{i}{\arg\max}F_{i} \end{array}$$

#### 3.2.2. Estimate the Forward Derivative and Saliency Map

In black-box attack, as we cannot access the full knowledge of the model, we cannot compute the forward derivative directly, so we consider another method to acquire it. We estimate the forward derivative $\lambda_{cij}$ of super-pixels $\psi_{ij}$ within key frame $x_{i}^{*}$ based on NES as the contribution of that super-pixel to the output. Different from maximizing the expected value of the loss function [27], we maximize that of the output under the searching distribution $\pi (\theta |x_{i}^{*})$. For a frame $x_{i}^{*}$, we compute it as Equation (8) [15]:

(8) $$\nabla_{x_{i}^{*}}E_{\pi (\theta |x_{i}^{*})}\left[F\left(X\right)\right]=E_{\pi (\theta |x_{i}^{*})}\left[F\left(X\right)\nabla_{x_{i}}\log\left(\pi \left(\theta |x_{i}^{*}\right)\right)\right]$$

With a trick similar to [15], we choose the antithetic searching distribution of random Gaussian noise around $x_{i}^{*}$ as $\pi (\theta |x_{i}^{*})$, where $\theta =x_{i}^{*}+\sigma B$, σ is a constant, and B has the same size as $x_{i}^{*}$. For sampling, we set $B_{o}∼N\left(0,I\right)$, $o\in \{1,2,\cdots ,\frac{T}{2}\}$. And it sets $B_{s}=-B_{T-s+1}$, where $s\in \{\left(\frac{T}{2}+1\right),\cdots ,T\}$. Each element of $B_{o}$ and $B_{s}$ are defined as $b_{oj}$ and $b_{sj}$, respectively, j∈{1,2,⋯,M}, and *M* is the number of super-pixels of $x_{i}^{*}$. Lastly, the forward derivative of key frame $x_{i}^{*}$ can be estimated with Equation (9):

(9) $$\nabla_{x_{i}}F\left(X\right)=\frac{1}{M\sigma }\sum_{j=1}^{M}\delta_{j}F\left(x_{i}+\sigma \delta_{j}\right)$$

(10) $$\lambda_{cij}=\frac{1}{T\sigma }\sum_{u=1}^{T}b_{uj}F_{c}\left(x_{i}^{*}+\sigma B_{u}\right)$$

The $b_{uj}$ is *j*th element of $B_{u}$. Similar to the white-box attack, we then construct a saliency map $S_{ij}$ of the key frame $x_{i}^{*}$.

#### 3.2.3. Search for Key Pixels

In white-box attack, we change *n* when the algorithm drops into the “endless loop”, a trick that will automatically adjust the key frame’s index. However, in black-box attack, we should change the key frame index manually. The reason is that the perturbations added to each key pixel are so small that it cannot change the contribution value $F_{i}$ of that frame with these modified pixels. Therefore, the algorithm will select the same key frame in the following iteration, and if we let that situation continue, the frame chosen will continue to be disturbed, which leads to more perceptible noise. Moreover, the selected frame will choose other pixels that contribute less to the output to disturb, decreasing the perturbation effect. Hence, we should change a frame and spread these perturbations over different frames. We implement that trick by setting an iteration boundary ende so that the algorithm will change the frame index when the iteration reaches that value.

Indeed, changing the index of the key frame manually by changing *n* can bring another advantage, that users can set different *n* according to different situations. For example, if an application scene focuses on the perturbation of each frame but does not constrain the number of disturbing frames, we can set *n* to be large, but if an application scene can only change fewer frames but has a higher tolerance for per-frame disturbances, we should set *n* to be minor but the boundary value of disturbed pixels maxp large and minp minor.

The whole algorithm in the targeted attack of a black-box attack is summarized in Algorithm 2. Parameters of a black-box attack are only two more than those of white-box. They are M and ende.

#### 3.2.4. Analysis of the Algorithm under the Black-Box Setting

This section analyzes the computational complexity and the implementation cost of the AVEs generation method under the black-box setting. In the algorithm’s loop under the black-box setting, the key step uses SLIC for the key frames and estimates the forward derivative of the super-pixels of the key frame. According to the work [14] and the work [15], the complexity of SLIC and NES algorithms are O(n) and O(q×m), respectively. *n* is the number of objects that are needed to calculate super-pixels, the *q* is the number of the forward derivatives, and the *m* is the sampled points to help estimate forward derivatives. In that algorithm, the number of pixels that are needed to calculate super-pixels is H×W, the super-pixels of a key frame, and the number of the forward derivatives is (H×W)/ε, which is the number of super-pixels. Because the maximum number of the loop is maxe, the worst complexity is O(maxe×((W×H)/ε+m×(H×W)/ε), and the best complexity is O((W×H)/ε+m×(H×W)/k). According to the definition of infinite frequency, the complexity can be simplified as O(m×(H×W)/ε).
**Algorithm 2** Crafting the Black-Box Attack in the Target Mode**Input**X,F(·)**Input Parameter**g,C,y,maxe,n,maxp,η,minp,M,ende**Output**$X_{adv}$1:  Let $X_{adv}\leftarrow X$, $X^{*}=\left\{\right\}$, iter=1.2:  Set the target label g≠y.3:  **while** $F(X_{adv})\neq g$ and iter≠maxe **do**4:     **for** i←1 to *C* **do**5:       $M_{i}=1$6:       $F_{i}=F_{g}\left(X_{adv}\times mask\right)$.7:     **end for**8:     $x_{i}^{*}\leftarrow \underset{i}{\arg\max}F_{i}$.9:     **if** $x_{i}^{*}\notin X^{*}$ **then**10:       Add $x_{i}^{*}$ to the set of key frame $X^{*}$.11:   **end if**12:   Reduce the dimension of $x_{i}^{*}$ using SLIC: $x_{i}^{*}\leftarrow SLIC\left(x_{i}^{*}\right)$13:   Estimate forward derivative $\nabla_{x_{i}^{*}}F\left(x_{i}^{*}\right)$ by Equation (10).14:   Construct $S_{ij}$ for key frame $x_{i}^{*}$ by Equation (4).15:   Select super-pixels with top *n* value of $S_{ij}$ as key super-pixels $\psi_{ij}^{*}$ of key frame $x_{i}^{*}$.16:   Select *n* key pixels $p_{ijk}^{*},k\in \left[1,n\right]$ within $\psi_{ij}^{*}$ of $x_{i}^{*}$.17:   $p_{ijk}^{*}=p_{ijk}^{*}+\eta sgn\left(\lambda_{gij}\right)$.18:   **if** all key pixels ∉[minp,maxp] or iter/ende==0 **then**19:     $F_{i}=0$.20:       n←n×2.21:   **end if**22:   iter←iter+123:**end while**24:**return**$X_{adv}$

However, the algorithm under the black-box setting needs to query the DNNs model frequently. Therefore, in our paper, we use the metric queries to evaluate the time complexity of that algorithm. The detailed results are shown in Section 4.1.3.

## 4. Experiments

In this section, we use two state-of-the-art video attack methods to compare with our proposed method on two video threat models with two widely used datasets. We also show the performance of our method in different settings: black-box and white-box attacks. We focus on the overall perturbations, the number of the frames and pixels disturbed, and the time consumed in our experiment. A comprehensive evaluation of our method will be presented in this section.

### 4.1. Experimental Setting

#### 4.1.1. Datasets and Threat Models

We use two widely used datasets of video recognition: UCF101 [28] and HMDB51 [29], and two mainstream video recognition models: long-term recurrent convolutional networks (LRCN) [30] and C3D [31]. We use 16-frame snippets evenly sampled from each video as input. Table 1 summarizes the test accuracy with the two models. We randomly sample videos from UCF101 and HMDB51, which can be classified rightly, as the test video sample. We use Inception V3 [32] to extract features from frames and LSTM for video classification in LRCN.

#### 4.1.2. Metrics

- Fooling rate (FR): the percentage of adversarial videos that are successfully misclassified [19]. A larger value means a better attack.
- Perceptibility (mean absolute perturbation, MAP): the perceptibility score of an adversarial video, as $MAP=\frac{1}{Z}\sum_{k}\left|r_{k}\right|$, where *Z* is the total number of pixels in a video and $r_{k}$ is the perturbation added on the $k_{th}$ key pixel. A smaller value means better imperceptibility.
- Temporal sparsity (TS): the proportion of clean frames versus all frames of a video [9]. TS=1−L/T, where *L* is the number of perturbed frames, which is length of the set of the key frames $X^{*}$. A larger TS means better temporal sparsity.
- Spatial sparsity (SS): the proportion of clean pixels versus all pixels of a video. SS=1−PN/H, where PN is the number of perturbed pixels. A larger SS or a smaller PN means better temporal sparsity.
- Query (Q): in black-box attack, the number of queries of the threat model. A small value means fast attack.
- Time (Ti): in white-box attack, the time consumed when attacking a video. A small value means fast attack.

#### 4.1.3. Parameter Setting

**Figure 3:**
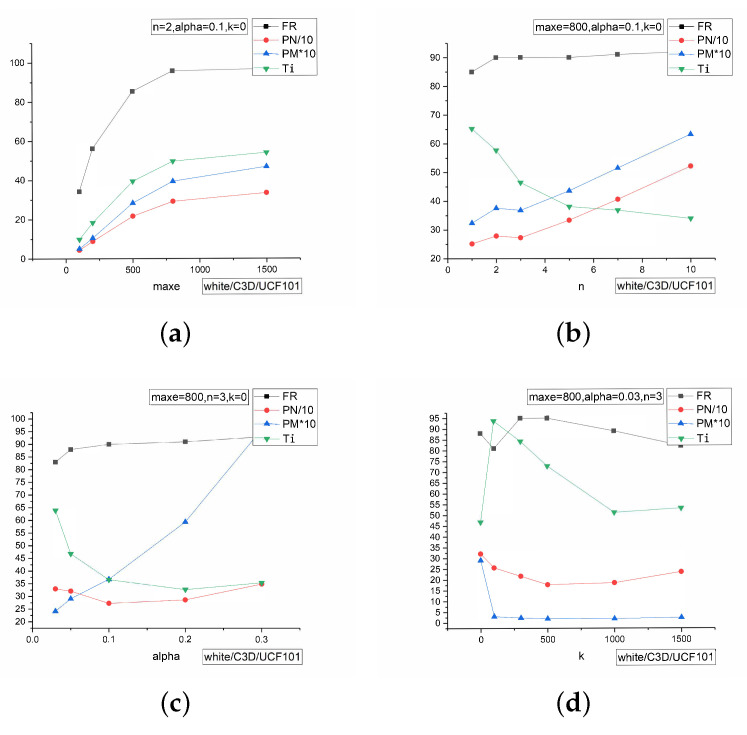
The result of different parameters. In order to keep uniform all metrics and keep the segment of *x*-axis consistent, we set PN/10 and PM*10. PN is the number of perturbed pixels and PM is short for MAP. All the performances are tested in white-box setting, C3D model with UCF101 dataset. (**a**) performances of different maxe; (**b**) performances of different *n*; (**c**) performances of different *ℵ*; (**d**) performances of different *k*.

In this section, we set the parameters used in two algorithms. We show the different performance of different parameters in Figure 3.

According to Figure 3, the first picture (Figure 3a) shows the results of different maximum iteration number maxe when keeping the number of key super-pixels n=2, the ratio of perturbations alpha=0.1, and the parameter of super-pixels k=0 constant. It shows that when the maxe is close to 800, the metrics of FR trend to be stable, while other metrics still have a little increase. Therefore, when maxe=800, it has a better performance. Then, we figure the difference of those metrics under different *n* with setting maxe=800 while other variables keep consistent as per Figure 3b. It shows that with *n* increases, the effect on FR of different *n* is small, but the other metrics will increase, and the line shows that n=3 is a tipping point. The trend is sharp after that line. Therefore, we set n=3 to have a better performance. The picture in Figure 3c is the difference of different *ℵ*, and the line ℵ=0.03 is the turning point. We pursue the higher FR and lower other metrics, so the appropriate value is ℵ=0.03. Figure 3d shows the differences under different *k* with the parameters selected above, and when k=500, it has an appropriate performance than other *k*. That analysis is under the normal pursuit of better FR, and when the pursuit is different, it can set different parameters. If the environments require less time, the pursuit of smaller Ti is the first, and then n=5 will perform better than n=3.

Above all, we know that there will be a better performance when maxe=800, n=3, and K=500 in HCF101, and K=1000 in HMDB51 initially; η=α×(maxp−minp), where α=0.03, and set maxe,minp as the maximum and minimum value of pixels in each frame, respectively, in white-box condition. In black-box condition, the difference is maxe=5000, $M={\left[0\right]}_{16}$, and ende=600.

### 4.2. Experimental Results

Table 2 shows the results of white-box attack. In the four experiments that were implemented under different models and datasets, we know that an adversarial video can be generated with only hundreds of pixels modified, and the fooling rate can be 100% in three of the results. When attacking C3D model with HMDB51, we find it is hard to attack. The fooling rate decreases to 96%, and the MAP and PN increase to 9.76 and 800.2, respectively, which are higher than others, obviously. We suppose that the features of it should be more robust [33]. Moreover, from the result, we can see that these metrics show the same trend of change, which correspond to the difficulty of adversarial attack of model and dataset. We found that the C3D model is more difficult to attack than LRCN, but the dataset of the different model shows a different trend. The HMDB51 dataset shows that it is more difficult to attack when the model is C3D, but it is easier to attack for LRCN.

Table 3 shows the result of black-box. Compared with white-box attack, we need thousands of pixels modified to attack the C3D model, and the PN of LRCN is also increased. The main reason is that the forward derivatives we estimated in the black-box setting are not accurate enough to search the real key pixels and frames. The estimated gradient is not completely consistent with the real gradient. The PN are increased; the reason is that the estimated forward derivatives are not the same as the real ones. Moreover, we can see that, although the other metrics show poor performance in the black-box attack, the TS is higher than the white-box attack. It benefits from the trick that the number of key frames can be adapted by ourselves according to the boundary of iterations that we set to change the index of key frame. In this experiment, we set it to ende=600 so that the maximum number of key frames is 9 and the minimum TS is 43.75%. Similarly, the trend of difficulty of the model and dataset is the same as that in the white-box attack. This feature belongs to the model and dataset themselves, whether generating adversarial videos in black-box attack or in white-box attack.

**Figure 4:**
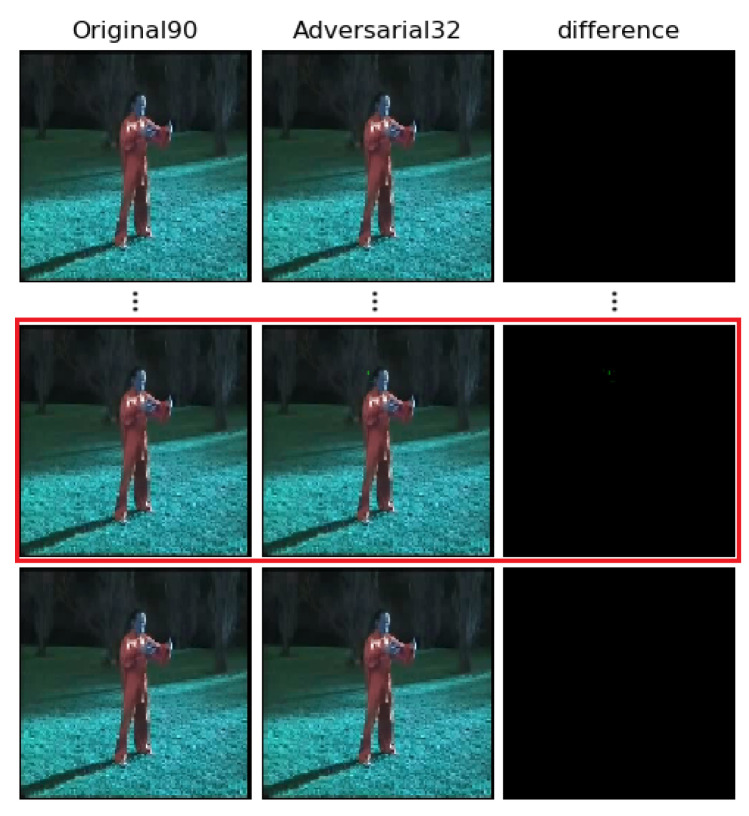
Other examples of adversarial frames generated by our algorithm in white-box adversarial attack. The clean video (**left**) can be classified correctly. The adversarial video (**middle**) with only one disturbed frame is misclassified, and the difference is shown as (**right**). The label 90 is “TaiChi”, while the label 32 is ‘GolfSwing’. The red square is the perturbed frame of that AVEs.

**Figure 5:**
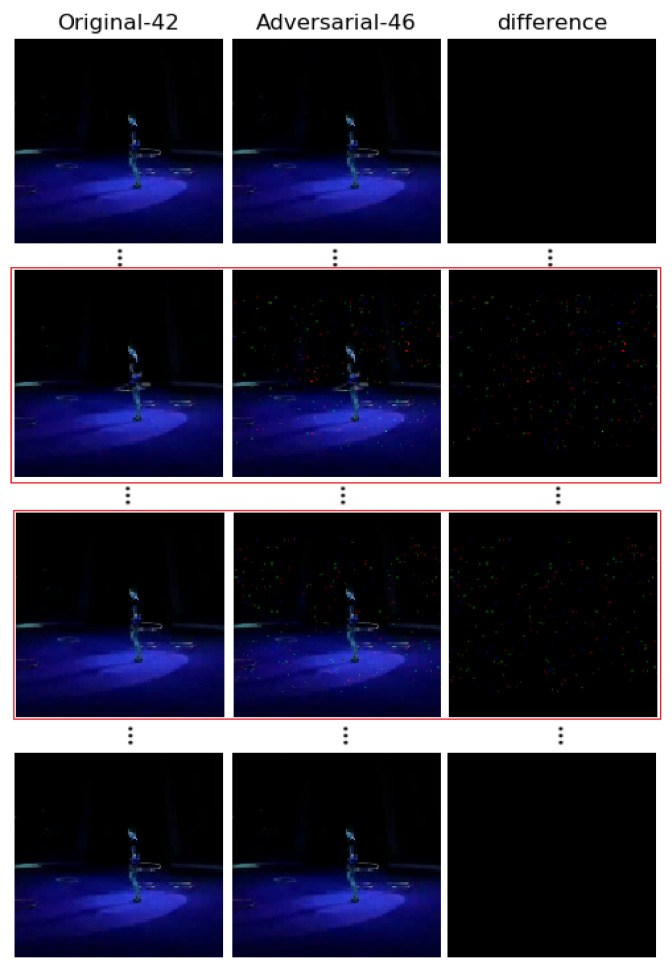
Other examples of adversarial frames generated by our algorithm in black-box adversarial attack. The clean video (**left**) can be classified correctly. The adversarial video (**middle**) with two disturbed frames is misclassified. The difference is shown as (**right**). The label 42 is “HulaHoop”, while the label 46 is ‘JumpingJack’. The red square is the perturbed frame of that AVEs.

Figure 4 and Figure 5 show the results of other adversarial videos generated by our algorithm. In Figure 4, it is generated in white-box attack. As shown, the original video with label 90 (TaiChi) can be misclassified by threat model into label 32 (GolfSwing) with only one frame perturbed, which is circled by a red line. Figure 5 shows the adversarial video generated in black-box attack, with two frames perturbed, which are circled by a red line, similarly. After being disturbed, the original video with label 42 (HulaHoop) can be misclassified into label 46 (JumpingJack). We notice a gap of disturbed pixels between the black-box and the white-box attack. It is obvious that the pixels that need to be perturbed in black-box attack are more than those in white-box attack. The main reason for that phenomenon is that there is also a gap between the real forward derivative and the estimated forward derivative. In other words, we cannot estimate the forward derivative absolutely.

### 4.3. Performance Comparison

In the white-box setting, we compared our method with the sparse attack [9] which used $l_{2,1}$-norm regularization-based optimization to generate sparse adversarial video. In the black-box setting, we compared our method with the V-BAD attack [11], which estimated the projection of an adversarial gradient on a selected subspace. The evaluations were performed on two video threat models with two public datasets.

Table 4 lists the different results of the sparse attack [9] and our method of white-box attack on LRCN model with two datasets. Table 5 shows the different results of the V-BAD attack [11] and our method of attacking LRCN model with two datasets in black-box setting. According to the evaluations, we have the following observations. In the white-box setting, our method improves the spatial sparsity of the attack, which means we can generate adversarial videos with fewer pixels modified, and we can generate an adversarial video faster than [9]. In the same temporal sparsity, our fooling rate is the same as [9]. Our method focuses on modifying fewer pixels to attack a model but these key pixels probably appear in different frames. In a word, these key pixels will spread over more frames, which leads to more frames perturbed and then a lower temporal sparsity sometimes. However, from the result, we can see that the temporal sparsity performs the same as other methods. Therefore, our method does not compensate spatial sparsity at the expense of temporal sparsity. From Table 5, in a black-box setting, our method can perform a temporally and spatially sparse attack with only few frames and pixels modified, and costing fewer queries. We have a better performance not only in temporal sparsity but also in spacial sparsity, and we improve the fooling rate to a new point.

## 5. Ablation Study

In this section, we test the difference between our algorithm with SLIC and that without SLIC. The results are shown in Table 6.

As Table 6 shows, the SLIC can help to decrease the time in the white-box attack and also decrease the queries of the model in the black-box attack. The SLIC can help to decrease the number of pixels that need to calculate the forward derivatives in the white-box setting and that to estimate the forward derivatives in the black-box setting. However, the SLIC also decreases the spatial sparsity (SS) under the different two settings. The reason is that the super-pixels share the same forward derivatives and they would be perturbed at the same time.

## 6. Conclusions

In this paper, we propose an algorithm to generate sparse adversarial videos in both black-box setting and white-box setting. In order to improve the temporal and spatial sparsity, we search for key pixels and key frames and only add perturbations on these selected pixels. In addition, to solve the problem of dimension explosion, we utilize super-pixels to decrease the number of pixels needed to be computed in white-box while estimated in black-box. Our algorithm can adapt to multiple video models and datasets. The experimental results show that our algorithm can attack a model with only <1% polluted pixels in a shorter time. However, though the sparse attack has many advantages, as discussed before, it cannot be generalized to the physical world. The reason is that it is hard to realize adding sparse perturbations in the physical world. In the future, we will explore the adversarial attack in the physical world to help to evaluate the robustness of the models.

## Figures and Tables

**Table 1:** Test accuracy of threat models.

Models	Datasets	
	UCF101	HMDB51
C3D	85.88%	59.57%
LRCN	64.92%	37.24%

**Table 2:** Experimental results of white-box attack.

Target Model	Dataset	Metrics				
		FR(%)	TS(%)	PN	Ti(s)	MAP
C3D	UCF101	100	25	211.5	29.5	2.67
	HMDB51	96	12.5	800.2	123.8	9.76
LRCN	UCF101	100	37.5	187.4	26.8	1.23
	HMDB51	100	50	170.8	32.5	1.70

**Table 3:** Experimental results of black-box attack.

Target Model	Dataset	Metrics			
		FR(%)	TS(%)	PN	Q
C3D	UCF101	88	43.75	3830.4	41584.9
	HMDB51	84	43.75	5940.3	52606.7
4LRCN	UCF101	84	81.25	437.3	19677
	HMDB51	95.9	81.25	263.8	14776

**Table 4:** Results of LRCN model on UCF101 and HMDB51 datasets in white-box setting.

Method	Metrics	FR(%)	TS(%)	SS(%)	Ti
Dataset					
UCF101	[9]	100	37.5	37.5	28.32
	Our	100	37.5	99.893	26.8
HMDB51	[9]	100	50	50	33.7
	Our	100	50	99.880	32.5

**Table 5:** Results of LRCN model with UCF101 and HMDB51 datasets in black-box setting.

Method	Metrics	FR(%)	TS(%)	SS(%)	Q
Dataset					
UCF101	[11]	76	0	0	40265
	Our	96	81.25	99.860	19677
HMDB51	[11]	90	0	0	24120
	Our	96	81.25	99.840	14776

**Table 6:** Results of the algorithm with SLIC and without SLIC. The “white” in the table means it is tested under the white-box setting and the “black” means it is tested under the black-box setting.

	Metrics	SLIC				No SLIC			
Dataset		FR(%)	TS(%)	SS(%)	Ti/Q	FR(%)	TS(%)	SS(%)	Ti/Q
UCF101	LRCN(white)	100	37.50	99.893	26.80	100	37.50	99.977	74.632
	LRCN(black)	84	81.25	99.860	19677	84	81.25	99.946	26674
HMDB51	LRCN(white)	100	50.00	99.880	32.5	100	50.00	99.979	127.6
	LRCN(black)	96	81.25	99.840	14776	94	81.25	99.968	15443

## Data Availability

Not applicable.
